# Development of a BMU-on-a-chip model based on spatiotemporal regulation of cellular interactions in the bone remodeling cycle

**DOI:** 10.1016/j.mtbio.2025.101658

**Published:** 2025-03-14

**Authors:** Sang-Mi Woo, Kyurim Paek, Yeo Min Yoon, Hyang Kim, Serk In Park, Jeong Ah Kim

**Affiliations:** a Center for Scientific Instrumentation, Korea Basic Science Institute, Daejeon, 34133, Republic of Korea; b Institute of New Horizon Regenerative Medicine, Myongji Hospital, Goyang, 10475, Republic of Korea; c Department of Biochemistry and Molecular Biology, Korea University College of Medicine, Seoul, 02841, Republic of Korea; d Department of Bio-Analytical Science, University of Science and Technology, Daejeon, 34113, Republic of Korea; e Chung-Ang University Hospital, Chung-Ang University College of Medicine, Seoul, 06973, Republic of Korea

**Keywords:** Human *in vitro* bone model, Basic multicellular unit, Tri-culture, Bone remodeling, Bone-on-a-chip

## Abstract

Bone remodeling is essential for maintaining bone homeostasis throughout life by replacing old bone with new tissue. This dynamic process occurs continuously within basic multicellular unit (BMU) through well-coordinated interactions among osteocytes, osteoblasts, and osteoclasts. However, a precise *in vitro* model that accurately replicates this mechanism has not yet been developed. In this study, we created a human *in vitro* BMU-modeling chip platform by tri-culturing cells within a chip unit integrated into a tissue culture well plate, enabling high-throughput three-dimensional (3D) cell culture. To establish the tri-culture, human osteoblasts were isolated from human surgical bone samples and differentiated into osteocytes within collagen gel inside the chip unit. Subsequently, osteoblasts and peripheral blood mononuclear cells (PBMCs) containing osteoclast precursors were added to the chip unit. To simulate each phase of the bone remodeling cycle, we optimized the tri-culture process by adjusting the timing and using two types of osteoblasts at different stages of differentiation. The completed tri-culture model successfully mimicked the bone formation phase. When receptor activators of nuclear factor kappa-Β (RANKL) and macrophage colony-stimulating factor (M-CSF) were introduced, the cells exhibited characteristics of the reversal phase, where osteogenic and osteoclastogenic environments coexist. Additionally, using more differentiated osteoblasts within the tri-culture platform induced osteoclast differentiation, resembling the bone resorption phase. Overall, our model effectively replicates each phase of the bone remodeling cycle in BMUs, both spatially and temporally. This advancement not only facilitates the study of the intricate mechanisms of bone remodeling and cellular function but also aids drug development by providing a robust bone model for testing target drugs.

## Graphical abstract

**Figure:**
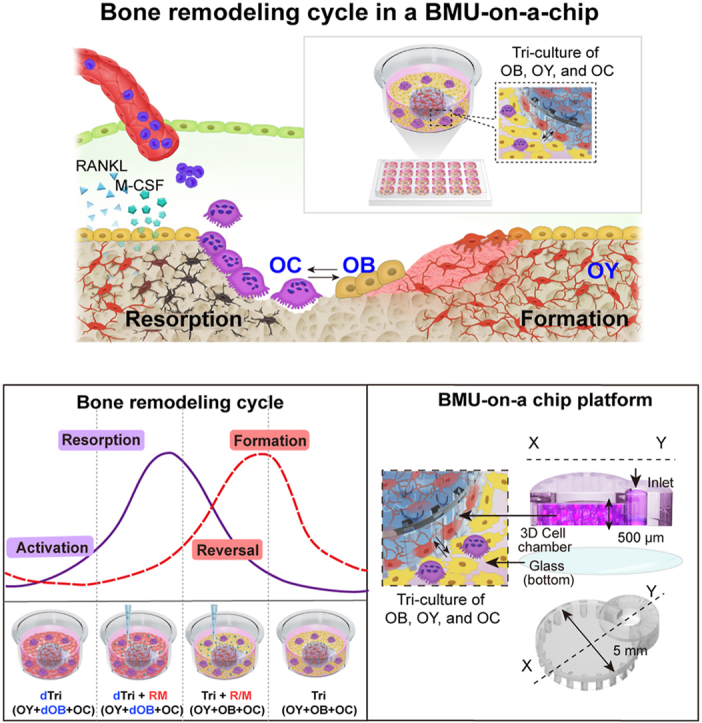

## 1 Introduction

Bones maintain their physiological balance through the bone remodeling cycle, which involves the removal of damaged or old bone and the formation of new bone. This cycle is tightly regulated by interactions among osteocytes (OYs), osteoblasts (OBs), and osteoclasts (OCs), which constitute the basic multicellular unit (BMU) [1,2]. Understanding bone biology, particularly the roles of these three cell types and the physiological functions regulated by their interactions, is crucial [3,4]. Therefore, developing advanced *in vitro* bone models that can replicate the bone remodeling cycle is essential. Such models can help elucidate the pathogenic mechanisms of bone-related diseases and provide valuable tools for effective drug development.

Recently, new *in vitro* bone models have been developed, incorporating advanced 3D technologies such as hydrogels, biochips, spheroids, cell sheets, scaffolds, and 3D printing [[5], [6], [7], [8], [9]]. Among these innovations, bone-on-a-chip (BOC) systems, based on microfluidic platforms that mimic bone, have been designed with careful consideration of the biological, biomechanical, and geometrical factors of bone [[10], [11], [12]]. One such BOC model successfully mimicked the lacunocanalicular structure by integrating biomechanical and geometric considerations in culturing primary human OBs within a microfluidic chamber filled with biphasic calcium phosphate microbeads [13]. Another study reported a bone remodeling model that recapitulated the bone remodeling cycle by co-culturing primary murine OBs and bone marrow mononuclear cells within a demineralized bone paper chip, incorporating chemical stimuli [14]. Additionally, bone remodeling has been studied through mechanical models. A study based on Wolff's law analyzed how external loading influences trabecular microstructure and density [15]. The finite element method (FEM) incorporated the lazy zone to assess loading frequency effects on remodeling [16]. Furthermore, a continuum model based on cellular activity suggested that moderate loading optimally enhances BMD [17]. These diverse research models have significantly advanced our understanding of bone mechanics, physiology, and the pathogenesis of bone diseases compared to traditional *in vitro* methods [11,18].

Despite these advancements, several challenges remain in fully replicating the complexities of bone biology. One key issue is the use of inappropriate cell sources in many models. Due to limitations in cell availability, most BOC models rely on animal cells or a mix of animal and human cells [10,19], which hinders the accurate replication of human-specific physiological mechanisms. Additionally, OYs, a critical cell type within the BMU, are often excluded or replaced with substitute materials in many BOC models due to the challenges associated with their culture [20,21]. A tri-culture model was proposed that includes human primary OBs, OYs, and OCs within a 3D transwell system, allowing for the observation of interactions among all three cell types in a spatially defined environment [22]. However, a fully developed *in vitro* tri-culture model capable of replicating the entire bone remodeling cycle, with all its complex steps, has not yet been reported.

The spatiotemporal complexity of the human bone remodeling cycle is still not fully understood [2,23]. This process occurs in different phases at various BMU sites within the human body, either asynchronously or simultaneously. The bone remodeling cycle is a hierarchical sequence of events organized into distinct phases, including activation, resorption, reversal, formation, and termination [2,24]. Therefore, it is crucial to observe and replicate specific temporal stages of the remodeling process. However, existing *in vitro* bone models primarily focus on either the bone resorption or bone formation phases [[25], [26], [27]].

In this study, we developed an *in vitro* human BMU-on-a-chip platform using a tri-culture system. Our platform, fabricated in a well plate format for high-throughput analysis, allows for easy monitoring of the entire bone remodeling cycle within a chip, with high uniformity. We established a human bone tri-culture system that models each phase of the bone remodeling cycle using human-derived cells. Human primary OBs were differentiated into OYs, and human peripheral blood mononuclear cells (PBMCs) were used as OC precursors. Additionally, chemical stimulants such as receptor activators of nuclear factor kappa-Β (RANKL) and macrophage colony-stimulating factor (M-CSF) were employed to induce paracrine effects for OC activation within the bone microenvironment. By optimizing the culture process for these three different cell types, we successfully recreated the bone remodeling cycle with its four key phases in this chip platform and confirmed that each phase was successfully simulated. This developed model can be utilized as a customizable tool to study various bone diseases arising from imbalances in bone metabolism. Furthermore, it offers the potential to determine the optimal timing and dosage of drug administration for specific phases of the bone remodeling cycle by elucidating the mechanisms of action of therapeutic agents.

## 2 Materials and methods

### 2.1 Fabrication of devices

The biochip, which incorporates a hydrogel unit within a well, was fabricated using polydimethylsiloxane (PDMS). The manufacturing process of these chips has been extensively detailed in our previous reports [[28], [29], [30]]. The fabrication process includes photolithography, punching, washing, sterilization, bonding, and assembly steps (Fig. S1). The bonding of the chip to the well plate was performed using air plasma treatment for 1 min at 100 W (Femto Science, Republic of Korea). To prevent shrinkage of the collagen gel, the surface of the assembled chip was coated with 1 mg/mL dopamine hydrochloride (Merck, Germany) in 10 μg/mL Tris buffer for 2 h.

### 2.2 Primary human OB isolation

The isolation of OBs followed established protocols [31,32]. Human bone tissues obtained from a patient were first cleared of soft connective tissue and then rinsed in sterile Dulbecco's phosphate-buffered saline (DPBS). Cancellous bone was extracted using sterile bone rongeurs and diced with scissors. The diced bone chips were then transferred to DPBS and sequentially washed using vortexing. The cleaned bone chips were cultured as explants at a density of 0.2 g of tissue/100 mm cell culture dish in high-glucose Dulbecco's Modified Eagle's Medium (DMEM; Cytiva, USA) supplemented with 10 % v/v fetal bovine serum (FBS; Hyclone, USA), 100 U/mL penicillin, and 100 μg/mL streptomycin (Hyclone, USA) in a 5 % CO_2_ incubator at 37 °C. After 2 weeks of explant culture, the OBs were seeded at a density of 5 × 10^3^ cells/cm^2^ and frozen for further experiments once they covered 70 % of the total surface area. This experiment was approved by the Institutional Review Board of Myongji Hospital (MJH 2020-09-025, MJH 2023-03-019) and complied with institutional ethical guidelines. Informed consent was obtained from patients undergoing total joint replacement surgery for degenerative musculoskeletal disorders.

### 2.3 Cell culture and differentiation

Normal human peripheral blood mononuclear cells (PBMCs) were purchased from Stemcell™ Technologies (USA). In this study, human OBs were cultured up to passage 6. Both human OBs and PBMCs were maintained in α-minimum essential medium (α-MEM; Gibco, USA) supplemented with 10 % v/v heat-inactivated FBS and 1 % v/v penicillin/streptomycin in a 5 % CO_2_ incubator at 37 °C. Human OBs were further differentiated into mature osteoblasts (dOBs) over 1–2 weeks in an osteogenic medium (OM) (Fig. S2). The OM consisted of complete α-MEM supplemented with 50 μg/mL L-ascorbic acid (Merck, Germany), 10 mM β-glycerophosphate disodium salt hydrate (Merck, Germany), and 100 nM dexamethasone (Merck, Germany). Human PBMCs were seeded at 1 × 10^5^ cells per well in a 24-well plate, considering the content ratio of monocytes (5–10 % of the total) [33] and differentiated into OCs for 1 week. The differentiation medium for PBMCs contained 50 ng/mL RANKL (R&D Systems, USA) and 25 ng/mL M-CSF (R&D Systems, USA) in OM (Fig. S6A). The medium for all cell cultures was refreshed every 3 days.

### 2.4 Co- or tri-culture

Primary human OBs were differentiated into OYs over 2 weeks under 2D culture conditions, after which the cells were embedded in 2 mg/mL rat tail collagen type-1 (Corning, USA) for an additional week. The collagen and 2-week differentiated OB mixture was then loaded into the chip and gelated at 37 °C for 30 min in a 5 % CO_2_ incubator, a process that takes approximately 3 weeks in total. For co- or tri-culture, OBs, dOBs, or OCs were seeded outside the chip in the well immediately after embedding the OYs, and the cells were cultured for 1 week. The seeding densities were 1 × 10^4^ cells for OYs, OBs, and dOBs, and 1 × 10^5^ cells for OCs. Osteogenic medium was used throughout the process, with medium changes every three days. The R/M groups were cultured in osteogenic medium supplemented with 50 ng/mL RANKL and 25 ng/mL M-CSF.

### 2.5 Enzyme-linked Immunosorbent assay (ELISA)

In the co-culture system, the conditioned medium (CM) was centrifuged at 2000 rpm for 5 min, and the supernatant was collected for ELISA. The concentrations of sclerostin (SOST) and interleukin-6 (IL-6) proteins in the CM were measured using SOST (Abcam, UK) and IL-6 (R&D Systems, USA) ELISA kits. For SOST detection, the CM was concentrated 50-fold using Vivaspin® Turbo 4 with a 10 kDa molecular weight cutoff (Sartorius AG, Germany). Subsequent steps were conducted according to the manufacturer's protocol. All samples were analyzed using a microplate reader (SpectraMax^Ⓡ^ M4; Molecular Devices, USA) at 450 nm.

### 2.6 Quantitative reverse transcription polymerase Chain reaction (qRT-PCR)

Total RNA from human cells was extracted using the RNeasy mini kit (Qiagen, Germany) according to the manufacturer's instructions. RNA concentration and quality were assessed using a NanoDrop™ 2000 Spectrophotometer (Thermo Fisher Scientific, USA). The extracted RNA was then reverse-transcribed into cDNA using the ReverTra Ace™ qPCR RT kit (Toyobo, Japan). qRT-PCR was performed in three steps (denaturation at 95 °C, annealing at 60 °C, and extension at 72 °C) using a miScript SYBR Green PCR kit (Applied Biosystems, USA). Each step was performed for 30 s and was repeated for 40–50 cycles using a PCR machine (QuantStudio 3; Thermo Fisher Scientific, USA). qRT-PCR was employed to quantify mRNA levels of markers associated with bone remodeling. The markers detected in embedded OYs included dentin matrix acidic phosphoprotein 1 (*DMP1*), sclerostin (*SOST*), receptor activator of nuclear factor kappa-Β ligand (*RANKL*), osteoprotegerin (*OPG*), and fibroblast growth factor 23 (*FGF23*), which are known OY differentiation markers. For OBs, the markers included runt-related transcription factor 2 (*RUNX2*), collagen-1α (*COL-1α*), alkaline phosphatase (*ALP*), osteocalcin (*OCN*), and bone morphogenetic protein 2 (*BMP2*), known OB differentiation markers. Additionally, monocyte chemoattractant protein 1 (*MCP1*), cathepsin K (*CTSK*), tartrate-resistant acid phosphatase (*TRACP*), and matrix metalloproteinase 9 (*MMP9*), known OC differentiation markers, were measured in OBs or OCs. The primers used for these analyses are listed in Table S1.

### 2.7 Immunocytochemistry

The cells were first fixed with 4 % paraformaldehyde (Merck, Germany) and then permeabilized with DPBS (Gibco, USA) containing 0.5 % v/v Triton X-100 for 10 min. Following permeabilization, blocking was carried out using 10 % v/v FBS in DPBS for 60 min. F-actin was stained with Phalloidin 594 (Invitrogen, USA) at a 1:400 dilution in blocking solution and incubated overnight at 4 °C in the dark. For nuclear staining, Hoechst 33342 (Thermo Fisher Scientific, USA) was diluted 1:1000 in blocking solution and incubated for 10 min at room temperature. Each step was followed by three washes with DPBS. Fluorescence images were captured using a fluorescence microscope (Celena X; Logos Biosystems, Republic of Korea).

### 2.8 TRACP & ALP activity measurement

The activities of TRACP and ALP were measured using a TRACP & ALP assay kit (Takara, Japan) and a TRACP & ALP double-stain kit (Takara, Japan). All experiments were conducted according to the manufacturer's instructions. Co-culture or tri-culture cells were washed twice with DPBS. For the assay or staining, cells were either lysed with the provided extraction solution or fixed with a solution of ethanol and acetone (1:5). The assay procedure involved the following steps: TRACP buffer was prepared by mixing acid phosphatase buffer and sodium tartrate solution in a 1:10 ratio. The lysate was then mixed with the TRACP or ALP buffer in a maximum1:1 ratio, respectively. The mixture was incubated at 37 °C for 15–60 min, after which a stop solution was added. TRACP or ALP activity was measured using a microplate reader (SpectraMax^Ⓡ^ M4; Molecular Devices, USA) at 405 nm. For staining, the ALP and TRACP substrate solutions were used as provided. To detect the tartrate-resistant enzyme, sodium tartrate (0.1 vol) was added to the TRACP substrate solution. Each substrate solution was applied to fixed cells and incubated at 37 °C for 15–60 min. After incubation, the solution was removed, and the cells were washed three times with sterile distilled water to stop the reaction. Images were then captured using a microscope (Celena X; Logos Biosystems, Republic of Korea).

### 2.9 Cell proliferation assay

OBs were seeded at a density of 1 × 10^4^ cells and co-cultured with embedded OYs for one week, either in a transwell or within the chip platform. To assess cell viability, the OBs were treated with 50 μL of water-soluble tetrazolium salt (Donginbiotech, Republic of Korea) per well and incubated for 60 min at 37 °C. Cell activity was then measured at 450 nm using a microplate reader (SpectraMax^Ⓡ^ M4; Molecular Devices, USA).

### 2.10 CTSK activity assay

CTSK activity was measured using a fluorescence-based assay kit (Abcam, UK) according to the manufacturer's protocol. Cells were lysed with cathepsin K cell lysis buffer and incubated on ice for 10 min. The cell lysates were then centrifuged at 12,000 rpm for 5 min, and the supernatant was transferred to a new tube. A total of 50 μL of the cell lysate was added to a 96-well plate, followed by an additional 50 μL of the prepared reaction buffer. The provided substrate was then added at a final concentration of 200 μM, and the mixture was incubated at 37 °C for 60 min. Enzyme activity was recorded using a microplate reader (SpectraMax^Ⓡ^ M4; Molecular Devices, USA) at Ex 400/Em 505 nm.

### 2.11 Statistical analysis

All experiments were performed in triplicate at a minimum. The data are presented as the mean ± standard deviation (SD). Statistical significance was determined using the Student's *t*-test. Significance levels are indicated as follows: ∗ or #*p* < 0.05, ∗∗ or ##*p* < 0.01, and ∗∗∗ or ###*p* < 0.001.

## 3 Results and discussion

### 3.1 BMU-on-a-chip platform

**Fig. 1:**
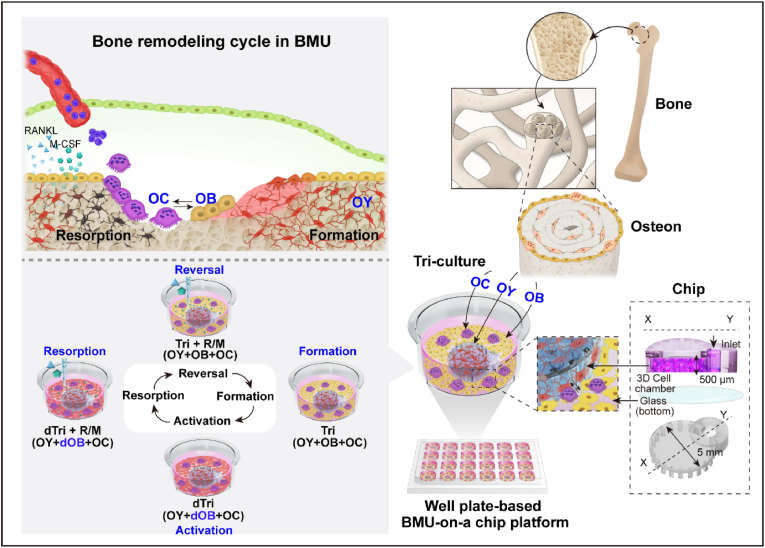
Schematic overview**.** The bone remodeling process occurs continuously within BMU, which consist of OYs, OBs, and OCs. To simulate the bone remodeling cycle, which includes four phases (activation, resorption, reversal, and formation), the tri-culture system was optimized to recreate the consecutive phases of the bone remodeling cycle (left). This tri-culture system, resembling the osteon structure, was fabricated in a well-plate format to facilitate high-throughput drug testing (right). BMU: basic multicellular unit; OBs: osteoblasts; dOB: differentiated OB; OYs: osteocytes; OCs: osteoclasts; R/M: receptor activator of nuclear factor kappa-Β (RANKL) and macrophage colony-stimulating factor (M-CSF).

*In vivo*, the osteon is cylindrical and features a central Haversian canal surrounded by bone matrix, where OYs are embedded. OBs and OCs are situated on the bone surface (Fig. 1, top right) [34]. In our previous study, we developed an osteon-mimicking bone-on-a-chip using OBs and OYs within a hydrogel unit integrated into a well plate, effectively simulating the structural features of an osteon [29]. To further replicate the physiological properties of an osteon [35], it is essential to develop a BMU-on-a-chip platform that incorporates the three cell types responsible for BMU regulation: OYs, OBs, and OCs. The BMU is the functional unit of the osteon, critical for mimicking the bone remodeling cycle (Fig. 1, bottom right). This cycle comprises four phases—activation, resorption, reversal, and formation—each tightly regulated by interactions among these three cell types within the BMU (Fig. 1, top left) [2]. Our model introduces a tri-culture system that facilitates the investigation of intercellular interactions, such as the SOST and RANKL/OPG pathways, which are pivotal in regulating the bone remodeling cycle [4,36,37]. Prior to integrating these cells into the tri-culture system, each cell type underwent an optimization process. To further induce specific phases of bone remodeling within the BMU-on-a-chip, we introduced cytokines such as RANKL and M-CSF (R/M), which simulate the BMU environment. These cytokines synergistically promote the maturation of OC precursors and activate bone resorption [38,39]. Through these combined strategies, our model effectively recapitulates the microenvironmental characteristics of each phase of bone remodeling in a modular fashion (Fig. 1, bottom left).

### 3.2 Optimization of OY differentiation

**Fig. 2:**
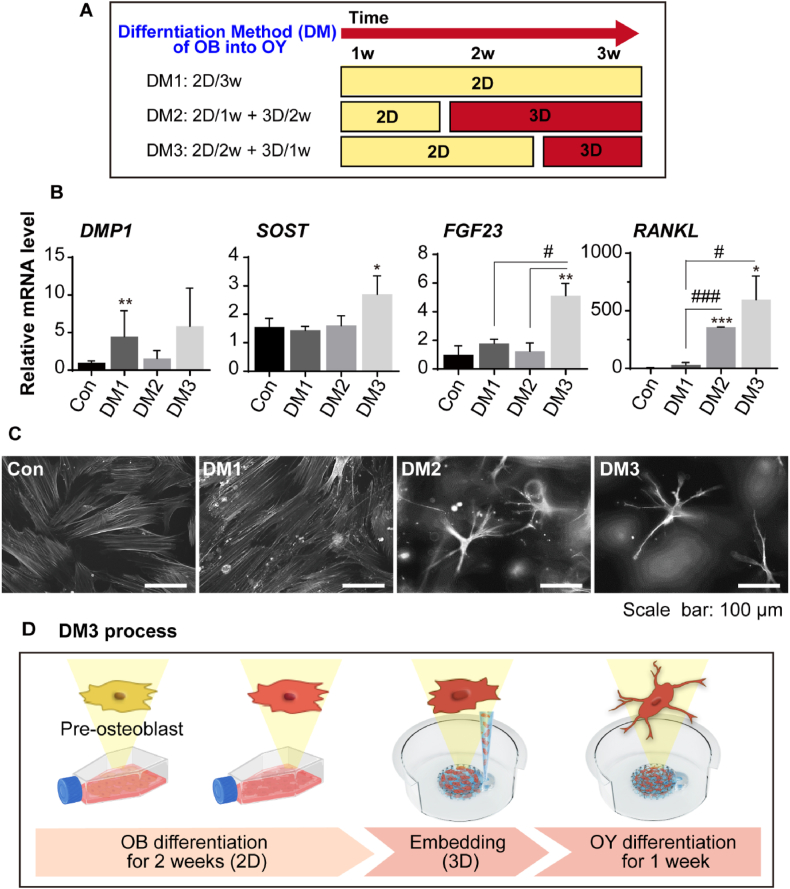
Optimization of OB differentiation into OYs. (A) Timeline for OB differentiation into OYs using three methods: DM1, 2D culture for 3 weeks (2D/3w); DM2, 2D culture for 1 week followed by 3D culture for 2 weeks (2D1w + 3D/2w); and DM3, 2D culture for 2 weeks followed by 3D culture for 1 week (2D/2w + 3D/1w). (B) Gene expression levels of OY markers (*DMP1*, *SOST*, *FGF23*, and *RANKL*) in control (Day 0), DM1, DM2, and DM3 groups, normalized to *GAPDH* (n = 2 or 3, ∗ vs. Con, # vs. DM3). (C) OY morphology in DM groups, with OYs stained using Phalloidin 594 (F-actin) after the differentiation process. (D) The final DM3 process selected for OB differentiation into OYs: OBs were cultured in osteogenic medium for 2 weeks in 2D, then embedded in 2 mg/mL of 3D collagen gel for 1 week. All data are expressed as mean ± SD (∗, #*p* < 0.05; ∗∗, ##*p* < 0.01; ∗∗∗, ###*p* < 0.001). *DMP1*: dentin matrix acidic phosphoprotein 1; *SOST*: sclerostin; *FGF23*: fibroblast growth factor 23; *RANKL*: receptor activator of nuclear factor kappa-Β ligand.

OYs are crucial regulators of bone remodeling, orchestrating the bone remodeling cycle through communication with OBs and OCs. However, their role has been relatively overlooked compared to OBs and OCs [40,41]. This is largely due to the difficulty of isolating OYs, as they are embedded within the bone matrix, making them challenging to study. Although advancements in isolation techniques and the use of alternatives like mesenchymal stem cell differentiation have been made, the supply and differentiation of OYs remain limited due to the complex and time-consuming processes involved [20,42]. In fact, at least 6 weeks are generally required for complete OY differentiation. However, a recent study reported a culture method that significantly shortened this period to 3 weeks by combining 2D and 3D cultures [21]. This method involved a one-week 2D culture followed by a two-week 3D culture, as depicted in DM2 of Fig. 2A. However, our findings indicate that our modified DM3 method, which consists of a two-week 2D culture followed by a one-week 3D culture in a chip, is more effective in promoting osteogenesis of OYs compared to DM1 or DM2 (Fig. 2B). Specifically, the gene expression levels of OY markers such as *SOST*, *FGF23*, and *RANKL* were significantly higher in the DM3 group. In contrast, there were no significant differences in *DMP1* levels among the groups, a marker typically associated with mineralization and used as an early OY or late OB marker. This suggests that OBs transitioned from late-stage OBs to mature OYs, expressing late markers like *SOST* and *FGF23* [43,44]. Additionally, *RANKL* expression increased as OBs differentiated into OYs [45], which is linked to OC activation and bone resorption [40,46]. As a result, osteocytic expression was confirmed to be highest in the DM3 group. This was also reflected in the morphological changes observed during differentiation, where undifferentiated OBs gradually transitioned into the sharp, star-shaped morphology characteristic of OYs (Fig. 2C) [47]. Notably, this star-shaped morphology was most pronounced in the DM3 group. Although incomplete star-shaped characteristics were also observed in OBs embedded in 3D culture from day 0 to three weeks, the shortened embedding period in the chip makes it more convenient to fabricate a complete tri-culture system (Fig. S3). Thus, the DM3 method was determined to be the most suitable for preparing OYs in the tri-culture system (Fig. 2D). However, from the perspective of the bone matrix, our new differentiation method involves only one week of 3D culture, which may not be sufficient for mineral accumulation. This issue could be improved in future studies through the application of advanced ECM, Nevertheless, the significant reduction in model fabrication time facilitates the rapid production and practical application of the *in vitro* model, further enhancing its value as an analytical tool for monitoring cell-cell interaction.

### 3.3 Performance of the BMU-on-a-chip

**Fig. 3:**
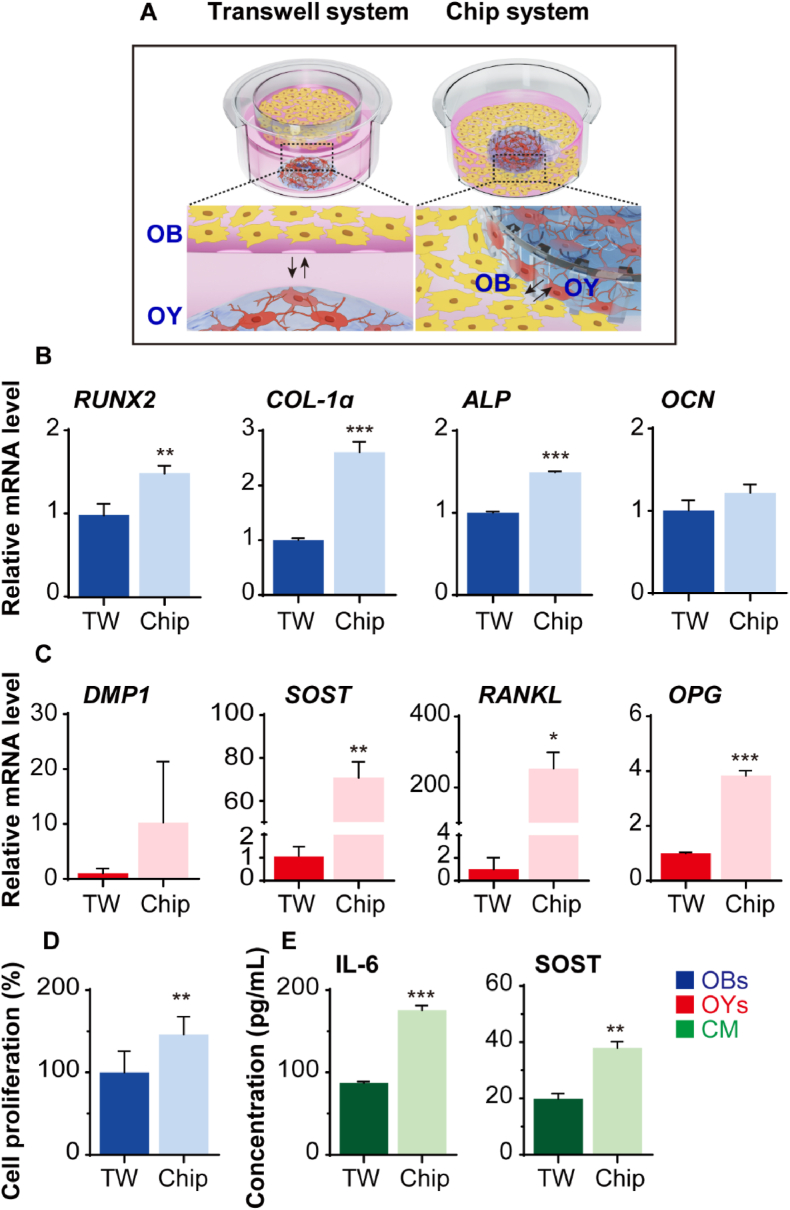
Comparative analysis of co-culture performance between the transwell (TW) system and the BMU-on-a-chip (Chip) system. (A) Schematic diagram of TW and Chip systems for OB and OY co-culture. In the TW system, cells interact indirectly through a vertically positioned membrane, whereas in the Chip system, cells interact directly at the chip interface. (B) Gene expression levels of OB markers (*RUNX2*, *COL-1α*, *ALP*, and *OCN*) and (C) OY markers (*DMP1*, *SOST*, *RANKL*, and *OPG*) after one week of co-culture. Cells were collected separately from each system, and values were normalized to *GAPDH* (n = 2 or 3). (D) Proliferation rate of co-cultured OBs over one week (n = 7). (E) Levels of IL-6 and SOST proteins in conditioned medium (CM) after one week of co-culture. Data are presented as mean ± SD (∗*p* < 0.05, ∗∗*p* < 0.01, ∗∗∗*p* < 0.001). *RUNX2*: runt-related transcription factor 2; *COL-1α*: collagen-1α; *ALP*: alkaline phosphatase; *OCN*: osteocalcin; *DMP1*: dentin matrix acidic phosphoprotein 1; *SOST*: sclerostin; *FGF23*: fibroblast growth factor 23; *RANKL*: receptor activator of nuclear factor kappa-Β ligand.

Traditionally, the transwell (TW) system has been widely used for co-culturing two or more cell types. The TW system employs a vertical setup where a porous membrane separates the cells within a well plate, allowing for indirect interactions. In our previous study, we demonstrated that our horizontally separated chip system, which allows two cell types to make direct contact at the border area, enhances cell-cell interactions, particularly in murine OB and OY cell lines (Fig. 3A) [29]. To assess the reproducibility of these results with human primary cells, we investigated whether our chip system outperforms the TW system in co-culturing primary human OBs and OYs. Both the chip and TW systems were subjected to identical culture conditions, including cell number, medium volume, gel volume, and a one-week co-culture period. We then compared the gene expression levels of OB and OY markers between the two systems. The results showed that the chip system significantly increased the expression levels of *RUNX2*, an early OB marker, as well as *COL-1α* and *ALP*, which are mid-stage OB markers, compared to the TW system. However, there was no significant difference in the expression of *OCN*, a late-stage OB marker (Fig. 3B) [46]. Additionally, OY markers such as *DMP1*, *SOST*, *RANKL*, and *OPG* exhibited an upward trend in the chip system (Fig. 3C) [48]. Moreover, OB proliferation was significantly enhanced in the chip system (Fig. 3D). The levels of IL-6, known to promote osteoclast formation [39], and the released SOST protein were also significantly higher in the CM from the chip system (Fig. 3E). These findings confirm that co-culture on our chip system is more favorable for primary bone cells, improving cell-cell interactions, proliferation, and differentiation compared to the TW system.

### 3.4 OY and OB co-culture in BMU-on-a-chips

**Fig. 4:**
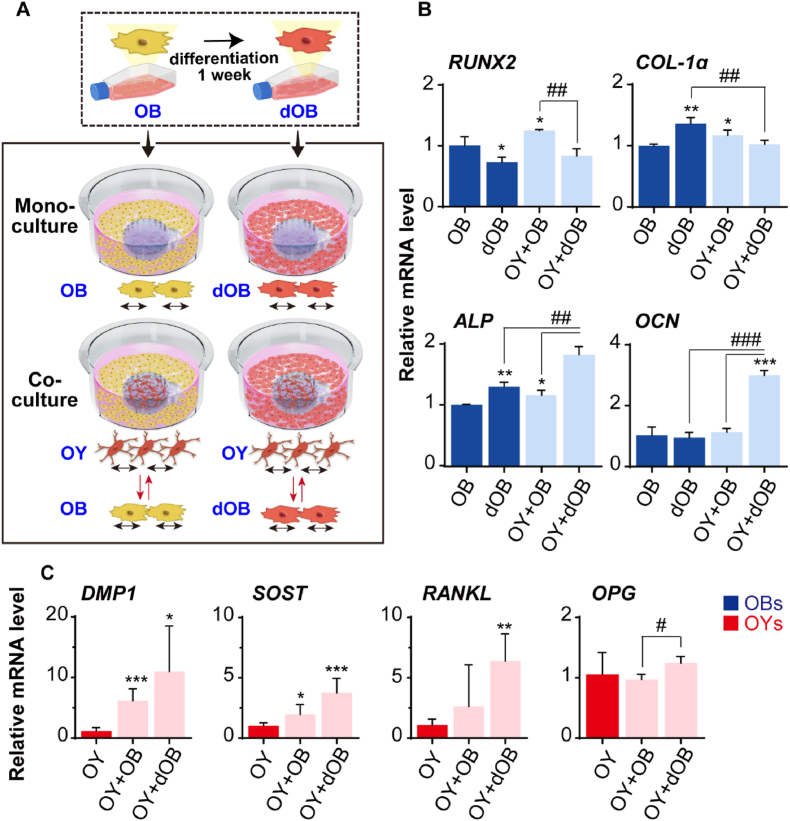
Effects of OB differentiation degree on osteogenesis during co-culture. (A) Two types of OBs were used: standard OBs and dOBs, which are OBs further differentiated for one week in a 2D osteogenic medium. Mono-culture groups consisted of OBs or dOBs cultured without OYs in the chip system, while co-culture groups involved OBs or dOBs co-cultured with OYs embedded in collagen gel within the chip for one week. OBs and OYs were collected separately for analysis. (B) Gene expression levels of OB markers (*RUNX2*, *COL-1α*, *ALP*, and *OCN*) and (C) OY markers (*DMP1*, *SOST*, *RANKL*, and *OPG*) were measured. Values were normalized to *GAPDH* (n = 3, ∗ vs. OB or OY, # vs. OY + dOB). All data are presented as mean ± SD (∗, #*p* < 0.05; ∗∗, ##*p* < 0.01; ∗∗∗, ###*p* < 0.001). *RUNX2*: runt-related transcription factor 2; *COL-1α*: collagen-1α; *ALP*: alkaline phosphatase; *OCN*: osteocalcin; *DMP1*: dentin matrix acidic phosphoprotein 1; *SOST*: sclerostin; *FGF23*: fibroblast growth factor 23; *RANKL*: receptor activator of nuclear factor kappa-Β ligand; *OPG*: Osteoprotegerin.

Previous studies have established that co-culturing OBs and OYs enhances bone formation [26,45,49]. However, the specific effects of OB differentiation levels on osteogenesis during co-culture have not been thoroughly investigated. To address this, we examined how the degree of OB differentiation influences osteogenesis by using more dOBs, which underwent an additional week of differentiation (Fig. 4A). In monocultures of OBs, the expression of *RUNX2* was high during the pre-OB stage but decreased as the cells matured. Conversely, *Col-1α* and *ALP* expression levels were low in the pre-OB stage, increased during the transition to OY, and then declined in later stages [42,50,51]. In the co-culture groups with OYs, markers for ALP and OCN were elevated. Notably, *OCN* expression significantly increased in the OY + dOB co-culture group. *OCN* is known to play a crucial role in promoting mineralization during the transition to the OY state [[52], [53], [54]]. By monitoring changes in these key markers, we demonstrated that using dOBs instead of less differentiated OBs enhances the maturation of the osteogenic environment. This effect was further amplified in the OY + dOB co-culture group (Fig. 4B). Additionally, OY markers were more strongly expressed in the OY + dOB group compared to the OY mono-culture group (Fig. 4C). We also explored the impact of extending OB differentiation for an additional two weeks during co-culture on osteogenesis (Figs. S4 and S5). The results showed a general decrease in osteogenesis markers, with a notable reduction in late-stage markers such as *SOST*, *FGF23*, and *OPG*. This provides insights into the terminal phase. However, further validation of OBs, which differentiate into OYs and bone lining cells, or undergo apoptosis [55,56], is needed to precisely identify the terminal phase. Due to limited knowledge of cell markers for this phase, aside from *SOST*, identifying the terminal phase remains challenging [3,57]. Thus, further studies on the termination phase are planned to be addressed in our future studies.

### 3.5 Tri-culture of OYs, OBs, and OCs in BMU-on-a-chips

**Fig. 5:**
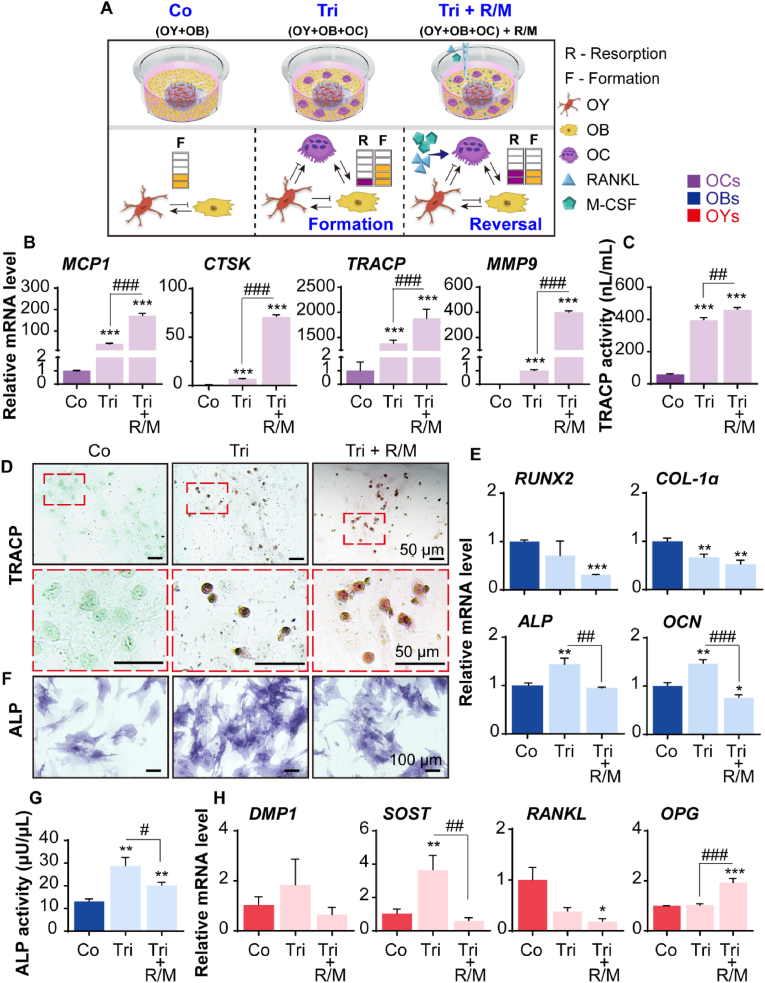
Effect of OC activity on osteogenesis in co-culture and tri-culture systems. (A) A schematic overview of the groups and the degree of interaction between cells. OBs were co-cultured with OYs or tri-cultured with OYs and OCs for 1 week. The Tri + R/M group refers to the RANKL (50 ng/mL) and M-CSF (25 ng/mL)-treated tri-culture group in osteogenic medium for 1 week. (B) Gene expression levels of OC markers (*MCP-1*, *CTSK*, *TRACP*, and *MMP9*). (C) TRACP activity of OCs in the co-culture or tri-culture system (n = 3). (D) Representative images of TRACP staining (reddish-purple) of OCs in co-culture or tri-culture systems. The enlarged image of the red dotted box is shown below. (E) Gene expression levels of OB markers (*RUNX2*, *COL-1α*, *ALP*, and *OCN*). (F) Representative images of ALP staining (purple) of OBs in co-culture or tri-culture systems. (G) ALP activity of OBs and OCs (n = 3). (H) Gene expression levels of OY markers (*DMP1*, *SOST*, *RANKL*, and *OPG*). The qRT-PCR results were normalized to *GAPDH* (n = 3). All data are expressed as mean ± SD (∗ vs. Co; # vs. Tri + R/M; ∗, #*p* < 0.05; ∗∗, ##*p* < 0.01; ∗∗∗, ###*p* < 0.001). R/M: receptor activator of nuclear factor kappa-Β (RANKL) and macrophage colony-stimulating factor (M-CSF); *MCP1*: monocyte chemoattractant protein 1; *CTSK*: cathepsin K; *TRACP*: tartrate-resistant acid phosphatase; *MMP9*: matrix metalloproteinase 9; *RUNX2*: runt-related transcription factor 2; *COL-1α*: collagen-1α; *ALP*: alkaline phosphatase; *OCN*: osteocalcin; *DMP1*: dentin matrix acidic phosphoprotein 1; *SOST*: sclerostin; *OPG*: osteoprotegerin.

PBMCs have been identified as a suitable source of osteoclast precursors [19]. Instead of isolating CD14^+^ monocytes from PBMCs, we applied the whole PBMCs directly to the model. The diverse immune cell population in PBMCs creates a microenvironment that more closely mimics *in vivo* microenvironment during osteoclast differentiation, thereby increasing the clinical relevance of the *in vitro* patient model [58]. Additionally, this approach eliminates the need for cell preprocessing, thereby improving accessibility for the application of multiple patient-specific models. For this reason, we incorporated PBMCs into a co-culture with OBs and OYs, thereby establishing a tri-culture system. To further enhance OC activity, R/M were added to the system. The concentrations of R/M used were based on previously reported studies, and cell fusion and multinucleation were observed after 3 days of treatment. OC differentiation was achieved by day 7 (Fig. S6B) [59,60]. We examined the effects of OC activity on osteogenesis under different culture conditions (Co, Tri, and Tri + R/M) within the co-culture and tri-culture systems (Fig. 5A). To assess the functionality of OCs within the tri-culture system, we analyzed the gene expression levels of OC markers, including *MCP1*, *CTSK*, *TRACP*, and *MMP9* (Fig. 5B), as well as TRACP enzyme activity (Fig. 5C–D). *MCP1*, which is involved in recruitment and cell-cell fusion [61], along with *CTSK*, *TRACP*, and *MMP9*, which are involved in bone matrix degradation, serve as indicators of OC activity and bone resorption [38,62]. The results demonstrated that OC differentiation activity was elevated in both the Tri and Tri + R/M groups compared to the Co group. Furthermore, in the Tri group, there was a significant increase in the expression of mid-to-late OB markers such as *ALP* and *OCN*, as well as the OY marker *SOST*, which is consistent with the mid-to-late bone formation phase [2,3]. Conversely, in the Tri + R/M group, bone formation markers decreased (Fig. 5E–G), while *OPG*, which inhibits bone resorption, significantly increased (Fig. 5H), indicating the onset of the bone reversal phase [3,4].

### 3.6 Activation of OCs by differentiation of OBs in BMU-on-a-chips

**Fig. 6:**
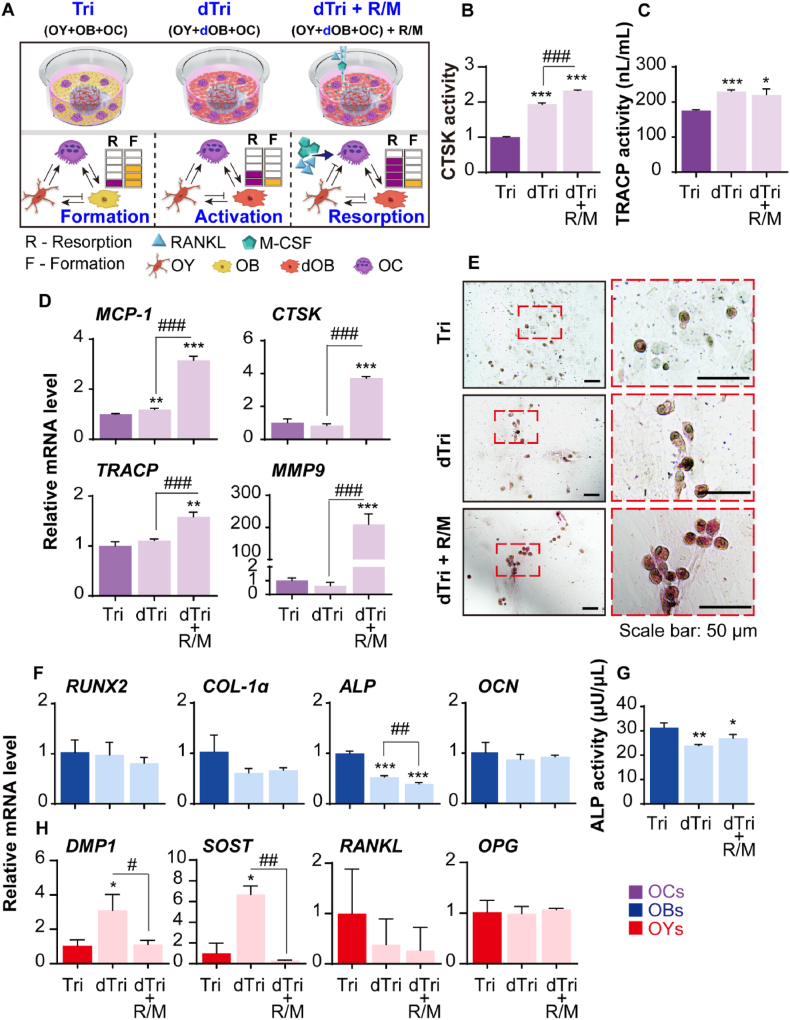
Effect of OB differentiation on OC activity within the tri-culture system. (A) A schematic overview of the groups and the degree of interaction between cells. dOBs are osteoblasts that have been further differentiated for 1 week in 2D in osteogenic medium. OBs or dOBs were tri-cultured with OYs and OCs for 1 week. The dTri + R/M group represents the tri-culture group treated with RANKL (50 ng/mL) and M-CSF (25 ng/mL) in osteogenic medium for 1 week. (B) CTSK activity and (C) TRACP activity of OCs (n = 3). (D) Gene expression levels of OC markers (*MCP-1*, *CTSK*, *TRACP*, and *MMP9*). (E) TRACP activity of OCs, visualized by reddish-purple staining. The dotted red box (left) indicates the enlarged area (right). (F) Gene expression levels of OB markers (*RUNX2*, *COL-1α*, *ALP*, and *OCN*). (G) ALP activity of OBs (n = 3). (H) OY markers (*DMP1*, *SOST*, *RANKL*, and *OPG*). qRT-PCR results were normalized to *GAPDH* (n = 3). All data are expressed as mean ± SD (∗ vs. Tri; # vs. dTri + R/M; ∗, #*p* < 0.05; ∗∗, ##*p* < 0.01; ∗∗∗, ###*p* < 0.001). R/M: receptor activator of nuclear factor kappa-Β (RANKL) and macrophage colony-stimulating factor (M-CSF); *MCP1*: monocyte chemoattractant protein 1; *CTSK*: cathepsin K; *TRACP*: tartrate-resistant acid phosphatase; *MMP9*: matrix metalloproteinase 9; *RUNX2*: runt-related transcription factor 2; *COL-1α*: collagen-1α; *ALP*: alkaline phosphatase; *OCN*: osteocalcin; *DMP1*: dentin matrix acidic phosphoprotein 1; *SOST*: sclerostin; *OPG*: osteoprotegerin.

The experiments shown in Fig. 4 suggest that the stage of osteogenesis can be modulated based on the degree of OB differentiation within the co-culture platform. Building on this, we introduced dOBs into the tri-culture platform, with or without the addition of R/M (Fig. 6A). The results indicated that the application of dOBs in the dTri group significantly increased OC activity (Fig. 6B–E) and the expression levels of OY markers *DMP1* and *SOST*, while the levels of OB markers remained relatively low or unchanged (Fig. 6F–G) compared to the Tri group, which mimics the bone formation phase. Notably, although OC differentiation activity was elevated in the dTri group, no significant changes were observed in the levels of *RANKL* and *OPG* (Fig. 6H). These findings suggest that the RANKL/OPG system, a key regulatory mechanism controlling OC differentiation and inhibition, which is modulated by hormonal stimuli such as 1α, 25(OH)_2_D_3,_ and PTH in OY, may not have been properly activated under direct RANKL treatment due to the absence of upstream signals [63]. However, overall, the dTri group appears to simulate a state akin to the activation phase, an early stage where OC differentiation and bone resorption are initiated without significant osteogenic activity [3]. When R/M was added to the culture, OC activity and bone resorption further increased compared to both the Tri and dTri groups (Fig. 6B–E), while osteogenic activity either decreased or remained unchanged (Fig. 6F-G). R/M is known to enhance bone resorption by regulating key factors involved in osteoclastogenesis, such as NFATc1, and promoting inflammatory osteolysis [64]. In the dTri + R/M group, the expression of OC markers increased without concurrent osteogenic activity (Fig. 6), indicating that this state closely resembles the resorption phase.

### 3.7 Summary of each phase setup to mimic the bone remodeling cycle

**Fig. 7:**
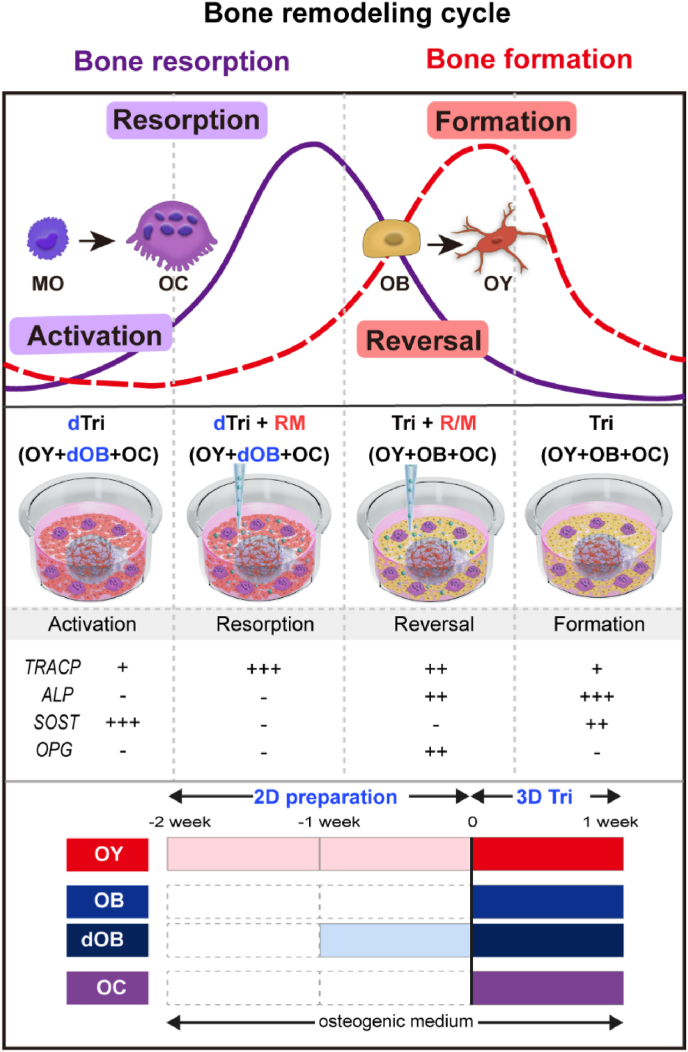
A schematic diagram summarizing the tri-culture system simulating each phase of the bone remodeling cycle. Bone remodeling cycle primarily consists of bone resorption and formation. Osteoclastogenesis begins with the 'activation' phase, after which OCs differentiate and become active in the 'resorption' phase. The transition from resorption to formation is termed the 'reversal' phase, followed by the 'formation' phase, where OBs differentiate. Additionally, morphological changes of cells occur at each phase. The BMU-on-a-chip platform, integrated with optimized culture and differentiation processes for OBs, OYs, and OCs, can mimic each phase of the bone remodeling cycle. Based on the mRNA expression levels shown in Fig. 5, Fig. 6, we have summarized the expression patterns of specific markers in the table. The 3D culture time is one week, excluding 2D cell preparation, and cells were maintained in osteogenic media throughout the entire culture period. MO: monocyte; OC: osteoclast; OB: osteoblast; OY: osteocyte; *TRACP*: tartrate-resistant acid phosphatase; *ALP*: alkaline phosphatase; *SOST*: sclerostin; *OPG*: osteoprotegerin.

The bone remodeling cycle progresses through distinct phases: activation, resorption, reversal, and formation. In this study, we demonstrated that these phases can be effectively simulated using a combinatorial experimental setup. First, we induced the bone resorption cycle (encompassing the activation and resorption phases) by utilizing more mature osteoblasts (dOBs) instead of younger OBs. Second, the addition of R/M effectively activated and sustained OC activity within the BMU-on-a-chip, facilitating the resorption and reversal phases. Additionally, it is important to consider the characteristic morphological changes of osteoclasts and osteoblasts at each phase and to focus on the changes in key markets. Based on the mRNA expression levels in Fig. 5, Fig. 6, we have summarized the changes in specific markers in the table. Notably, the entire culture process spans three weeks, which includes the 2D preparation of each cell type. However, the on-chip tri-culture process takes only one week, demonstrating that pre-optimization of the off-chip preparation can significantly reduce the time required for the more complex tri-culture process (Fig. 7).

Our approach addresses the limitations of existing models by allowing the bone remodeling cycle to be recapitulated at specific phases through spatiotemporal control. In addition, our model was fabricated by applying an equal cell ratio for the three cell types, which, although differing from the actual bone tissue cell population (OY: 90–95 %; OB: 4–6 %; OC: 1–2 %) [65,66], offers several advantages for studying cellular interactions [67]. By mainlining a balanced representation of cell types, it minimizes biases from population imbalances, enhances experimental reproducibility for more reliable cross-study comparisons, and simplifies data interpretation, resulting in clearer insights into fundamental cell-cell interactions.

We also discuss the scalability and limitations of our model from three perspectives: research, clinical, and industrial applications. In terms of research applications, this model enables spatiotemporal monitoring of marker expression trends, and the use of broader markers may allow for a more in-depth investigation of cell signaling mechanisms between bone cells (OY, OB, and OC). In clinical applications, this model can be customized for individual patients and used for personalized drug screening. It can also be applied to the study of correlation between specific diseases, and to bone metabolism disorders caused by remodeling imbalances. As for industrial applications, the well-plate-based chip is highly compatible with existing high-throughput equipment and systems and can be mass-produced for large-scale clinical drug testing.

However, our model also has several limitations. First, the mineral-enriched extracellular matrix of bone has not yet been used as a material for model fabrication, and although bone vasculature is an important component of bone structure, it has not been incorporated into this model. Second, in our model validation analysis, the limited use of cell markers restricts detailed mechanistic investigations, such as cell signaling. Additionally, the incomplete recreation of the terminal phase of the bone remodeling cycle needs to be addressed to enhance the completeness of the bone model. In conclusion, our future research aims to develop a more advanced model that not only realizes the scalability of our current model but also addresses its existing limitations.

## 4 Conclusions

In this study, we developed an advanced tri-cultured 3D BMU-on-chip system capable of mimicking the bone remodeling cycle. This model was designed to microscopically reproduce the fundamental physiological units of bone by tri-culturing OYs, OBs, and OCs within the integrated space of our chip. Beyond simply replicating the spatial characteristics of the bone osteon, this platform successfully reproduced the bone remodeling cycle by enhancing the physiological functions associated with cell-cell interactions. Our approach differed from previously reported bone remodeling models by employing combinatorial culture methods that integrate activation with chemical stimuli. This advanced model captures the complete sequence of the bone remodeling cycle (activation, resorption, reversal, and formation) successfully recapitulating the specific phases of bone remodeling that occur at focal sites within bone tissue. In summary, our approach provides a comprehensive spatiotemporal bone remodeling model, offering valuable insights into the entire bone remodeling cycle. While the model still has limitations, such as the lack of precise control over the early, mid, and late stages of each phase, its simplicity and reliance on cytokine-driven culture methods make the BMU model both accessible and reproducible. With the added advantage of high-throughput analysis capabilities, this platform holds significant potential for drug testing, studying diseases linked to imbalances in bone metabolism, and determining optimal timing for drug interventions.

## CRediT authorship contribution statement

**Sang-Mi Woo:** Writing – original draft, Visualization, Validation, Software, Methodology, Formal analysis, Data curation, Conceptualization. **Kyurim Paek:** Methodology, Conceptualization. **Yeo Min Yoon:** Methodology, Conceptualization. **Hyang Kim:** Writing – review & editing, Resources. **Serk In Park:** Writing – review & editing, Investigation. **Jeong Ah Kim:** Writing – review & editing, Writing – original draft, Supervision, Project administration, Investigation, Conceptualization.

## Declaration of competing interest

The authors declare the following financial interests/personal relationships which may be considered as potential competing interests: Sang-Mi WOO reports financial support from the 10.13039/501100003606Korea Foundation for Women in Science and Technology. Jeong Ah Kim reports financial support from the 10.13039/501100003725National Research Foundation of Korea and the 10.13039/501100003716Korea Basic Science Institute. We have permission from the journal to cite the data in Fig. S1. The other authors, if any, declare that they have no competing financial interests or personal relationships that could have appeared to influence the work reported in this paper.

## Data Availability

Data will be made available on request.
