# Supplementary material for: Development of a BMU-on-a-chip model based on spatiotemporal regulation of cellular interactions in the bone remodeling cycle
Source: Mater Today Bio. 2025 Mar 14;32:101658. doi: 10.1016/j.mtbio.2025.101658 (PMC11979395; doi:10.1016/j.mtbio.2025.101658)
Supplement: Multimedia component 1 [file mmc1.docx]

Supporting Information

**Development of a BMU-on-a-chip model based on spatiotemporal regulation of cellular interactions in the bone remodeling cycle**

Sang-Mi Woo ^a^ , Kyurim Paek ^a^, Yeo Min Yoon ^a^, Hyang Kim ^b^, Serk In Park ^c^, Jeong Ah Kim ^a ,d, e^ *

^a^ Center for Scientific Instrumentation, Korea Basic Science Institute, Daejeon 34133, Republic of Korea

^b^ Institute of New Horizon Regenerative Medicine, Myongji Hospital, Goyang 10475, Republic of Korea

^c^ Department of Biochemistry and Molecular Biology, Korea University College of Medicine, Seoul 02841, Republic of Korea

^d^ Department of Bio-Analytical Science, University of Science and Technology, Daejeon 34113, Republic of Korea

^e^ Chung-Ang University Hospital, Chung-Ang University College of Medicine, Seoul 06973, Republic of Korea

*Corresponding Author. Center for Scientific Instrumentation, Korea Basic Science Institute, Daejeon 34133, Republic of Korea

E-mail: [jakim98@kbsi.re.kr](mailto:jakim98@kbsi.re.kr)

Supplementary table:

Table S1. Sequence of human primer for qRT-PCR. The qRT-PCR experiment was performed with the following thermal cycling conditions: denaturation at 95 °C, annealing at 60 °C, and extension at 72 °C for 30 s, with 40 to 50 cycles for each step.

| **Gene** | **Forward** | **Reverse** |
| --- | --- | --- |
| ***RUNX2*** | TCTTAGAACAAATTCTGCCCTTT | GCTTTGGTCTTGAAATCACA |
| ***Col-1α*** | GTGCTAAAGGTGCCAATGGT | ACCAGGTTCACCGCTGTTAC |
| ***ALP*** | AAGCCGGTGCCTGGGTGGCCAT | ACAGGAGAGTCGCTTCAGAG |
| ***OCN*** | GCAGCTTGGTGCACACCTAG | GGAGCTGCTGTGACATCCAT |
| ***BMP2*** | GCCAAGCCGAGCCAACAC | CCCACTCGTTTCTGGTAGTTCTTC |
| ***SOST*** | ACCACCCCTTTGAGACCAAAG | GGTCACGTAGCGGGTGAAGT |
| ***RANKL*** | CGTTGGATCACAGCACATCAG | GTACCAAGAGGACAGACTCAC |
| ***OPG*** | CACTACTACACAGACAGCTGG | ACTCTATCTCAAGGTAGCGCC |
| ***DMP1*** | AGGAAGTCTCGCATCTCAGAG | TGGAGTTGCTGTTTTCTGTAGAG |
| ***FGF23*** | TGAGCGTCCTCAGAGCCTAT | TTGTGGATCTGCAGGTGGTA |
| ***MMP9*** | GTCACCTATGACATCCTGCAGTG | CTTTCCTCCAGAACAGAATACCAGTT |
| ***MCP1*** | TCGCGAGCTATAGAAGAATCA | TGTTCAAGTCTCGGAGTTTG |
| ***CTSK*** | TGAGGCTTCTCTTGGTGTCCATAC | AAAGGGTGTCATTACTGCGGG |
| ***TRACP*** | GACCACCTTGGCAATGTCTCTG | TGGCTGAGGAAGTCATCTGAGTTG |
| ***GAPDH*** | CTGGGCTACACTGAGCACC | AAGTGGTCGTTGAGGGCAATG |

*RUNX2*: runt-related transcription factor 2; *COL-1α*: collagen-1α; *ALP*: alkaline phosphatase; *OCN*: osteocalcin; *BMP-2*: bone morphogenetic protein 2; *SOST*: sclerostin; *RANKL*: receptor activator of nuclear factor kappa-Β; *OPG*: osteoprotegerin; *DMP1*: dentin matrix acidic phosphoprotein 1; *FGF23*: fibroblast growth factor 23; *MMP9*: matrix metalloproteinase 9; *MCP1*: monocyte chemoattractant protein 1; *CTSK*: cathepsin K; *TRACP*: tartrate-resistant acid phosphatase; *GAPDH*: glyceraldehyde 3-phosphate dehydrogenase.


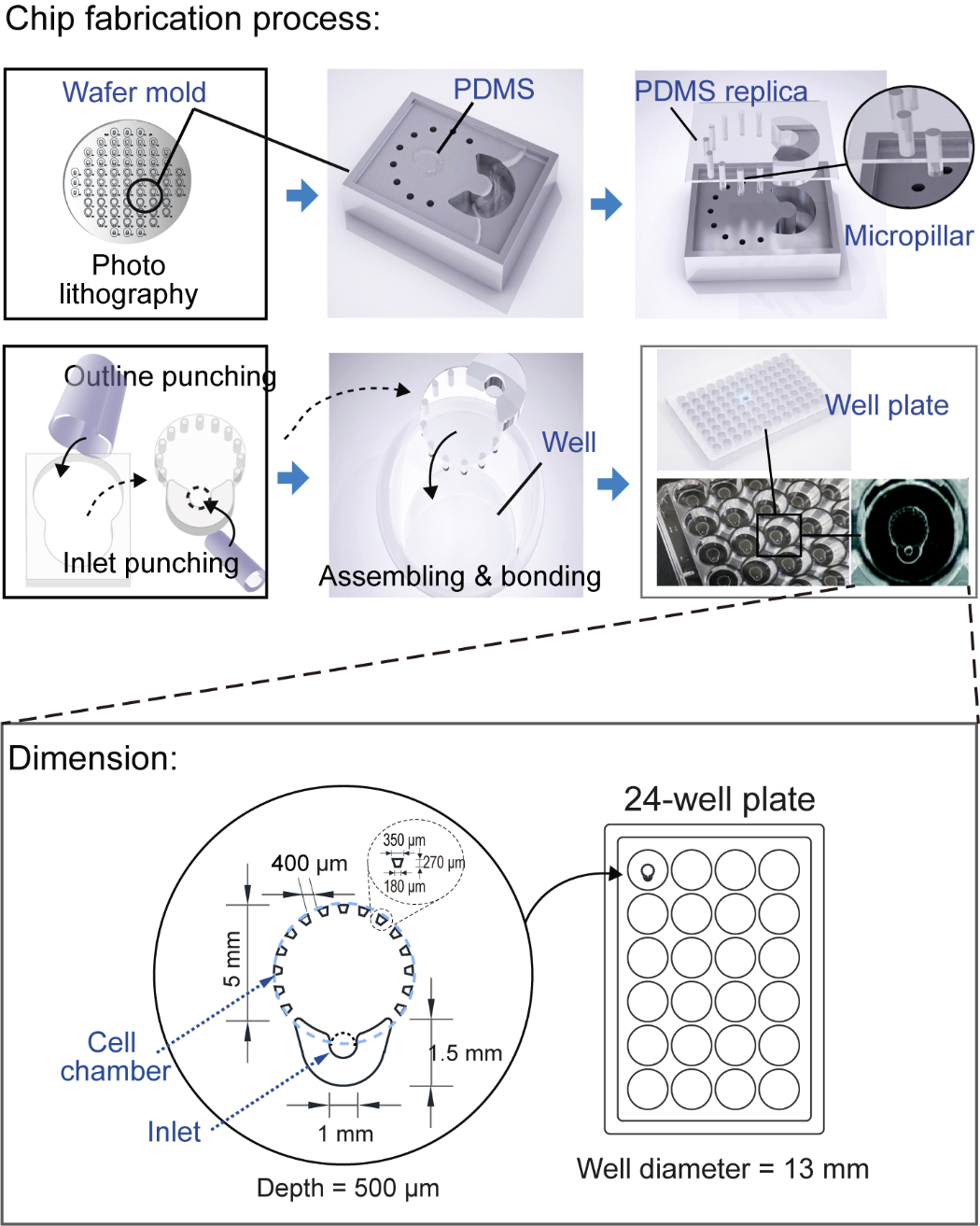
Supplementary figures:

Figure S1. Fabrication process and dimensions of the chip unit. The chip unit was fabricated using PDMS soft lithography and subsequently applied to a 24-well plate. The dimensions of the integrated chip unit within the well plate were slightly modified from the previous version. Reproduced with permission from ref. [1] CC BY 4.0 Copyright 2018, *Lab on a Chip* by the Royal Society of Chemistry.


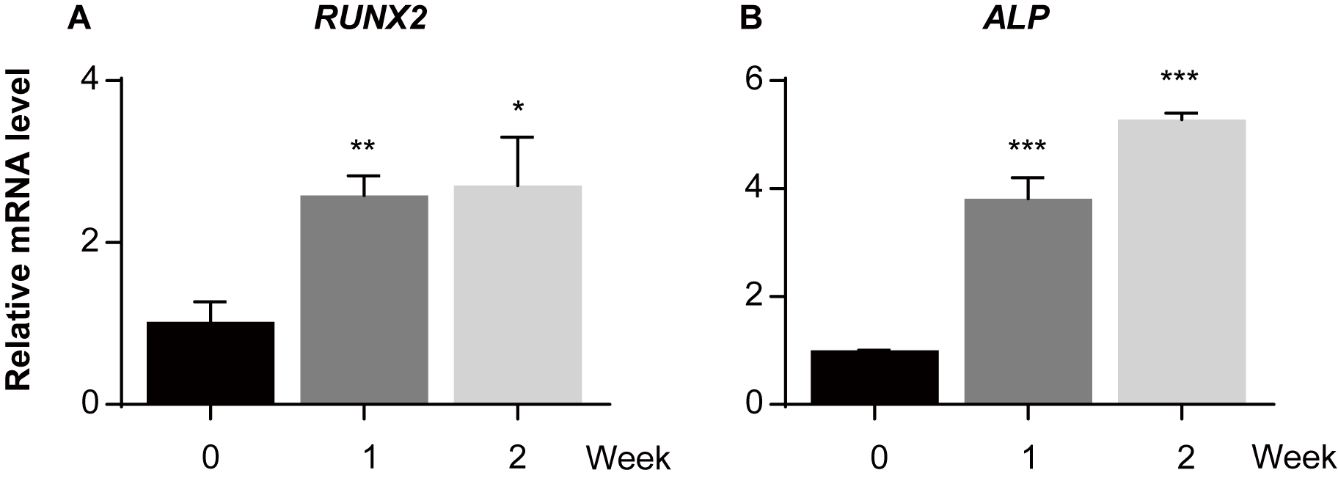
Fig. S2. Optimization of OB differentiation. (A and B) Gene expression levels of OB markers *RUNX2* and *ALP* were measured. OBs obtained from human bone were cultured in osteogenic medium for 0 to 2 weeks to promote further differentiation. The values were normalized to *GAPDH* (n = 3, * *p* < 0.05 vs. 0 weeks). All data are presented as mean ± SD (** *p* < 0.01, *** *p* < 0.001). *RUNX2*: runt-related transcription factor 2; *ALP*: alkaline phosphatase.


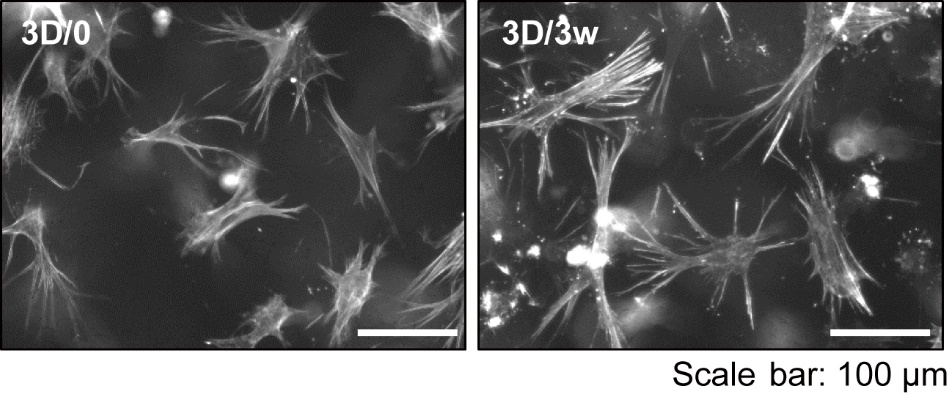
Fig. S3. Osteocytic phenotype of differentiated OBs in 3D collagen gel. OBs were embedded in a 2 mg/mL collagen gel and differentiated into osteocytes (OYs) over a 3-week period in osteogenic medium. The cells were stained with Phalloidin 594 to visualize F-actin and observed using a fluorescence microscope.


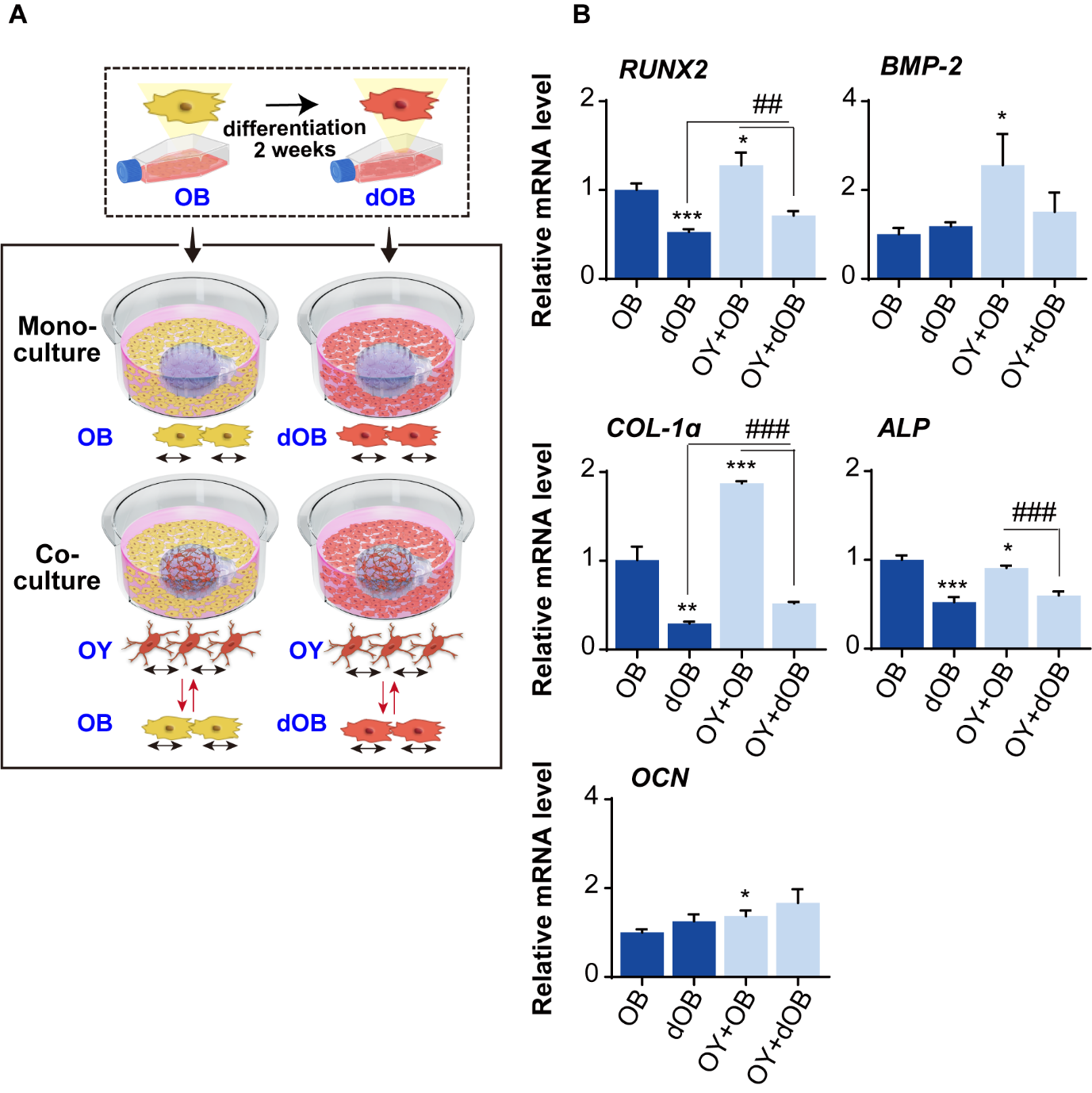
Fig. S4. Effect of 2-week differentiated OBs on osteogenesis during co-culture. (A) Two types of OBs were used: OBs and dOBs, the latter having undergone an additional 2 weeks of differentiation in 2D osteogenic medium. Mono-culture groups included OBs or dOBs without OYs in the chip system, while co-culture groups included OBs or dOBs co-cultured with OYs embedded in collagen gel for 1 week. OBs and OYs were collected separately for analysis. (B) Gene expression levels of OB markers *RUNX2*, *BMP-2*, *COL-1α*, *ALP*, and *OCN* were measured. The values were normalized to *GAPDH* (n = 3). All data are presented as mean ± SD (* *p* < 0.05 vs. OB; # *p* < 0.05 vs. OY+dOB; **, ## *p* < 0.01; ***, ### *p* < 0.001). *RUNX2*: runt-related transcription factor 2; *BMP-2*: bone morphogenetic protein-2; *COL-1α*: collagen-1α; *ALP*: alkaline phosphatase; *OCN*: osteocalcin.


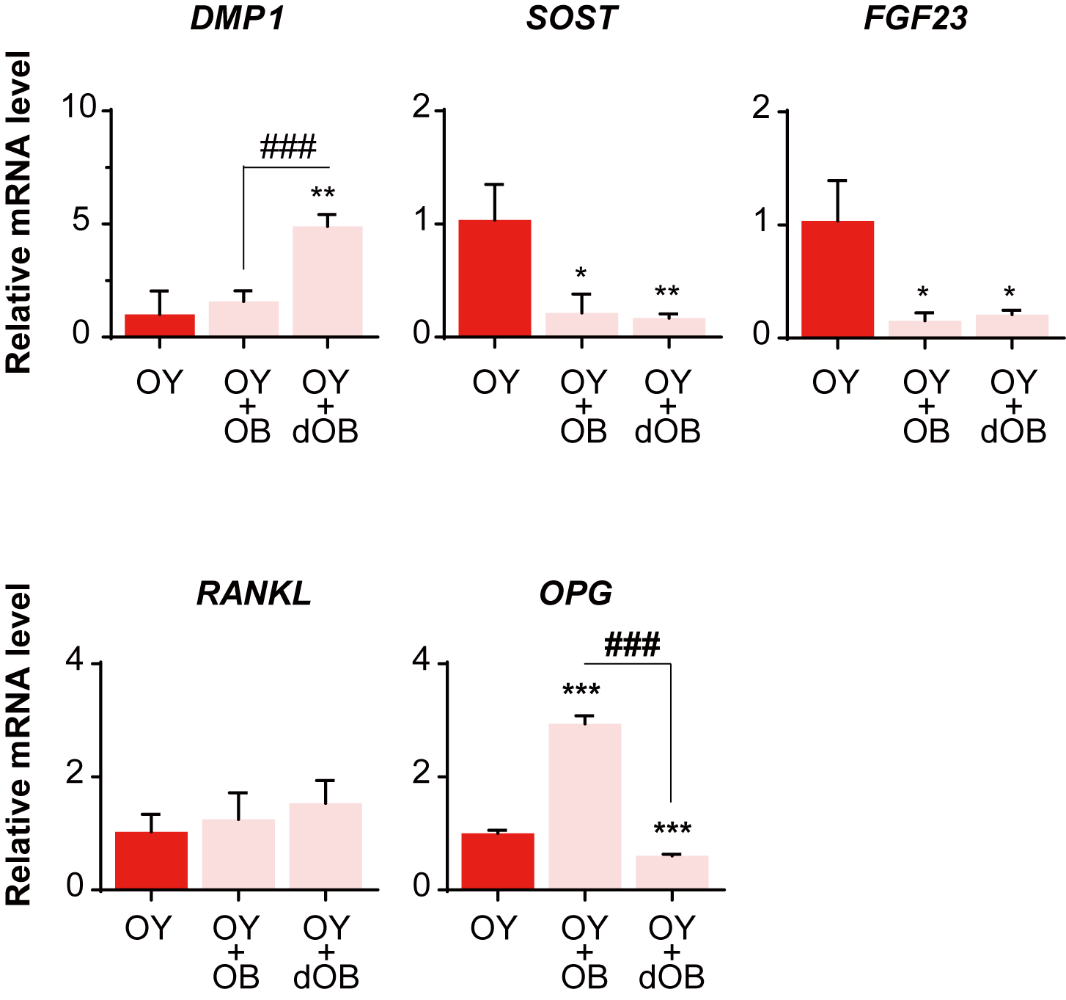
Fig. S5. Effect of 2-week differentiated OBs on osteogenesis of OYs during co-culture. dOBs were cultured for an additional 2 weeks in 2D osteogenic medium. OYs were embedded in a collagen gel and either mono-cultured or co-cultured with OBs or dOBs in osteogenic medium for 1 week. Gene expression levels of OY markers *DMP1*, *SOST*, *FGF23*, *RANKL*, and *OPG* were analyzed. The values were normalized to *GAPDH* (n = 3). All data are expressed as mean ± SD (* *p* < 0.05 vs. OY; # *p* < 0.05 vs. OY+dOB; **, ## *p* < 0.01; ***, ### *p* < 0.001). *DMP1*: dentin matrix acidic phosphoprotein 1; *SOST*: sclerostin; *FGF23*: fibroblast growth factor 23; *RANKL*: receptor activator of nuclear factor kappa-Β ligand; *OPG*: osteoprotegerin.


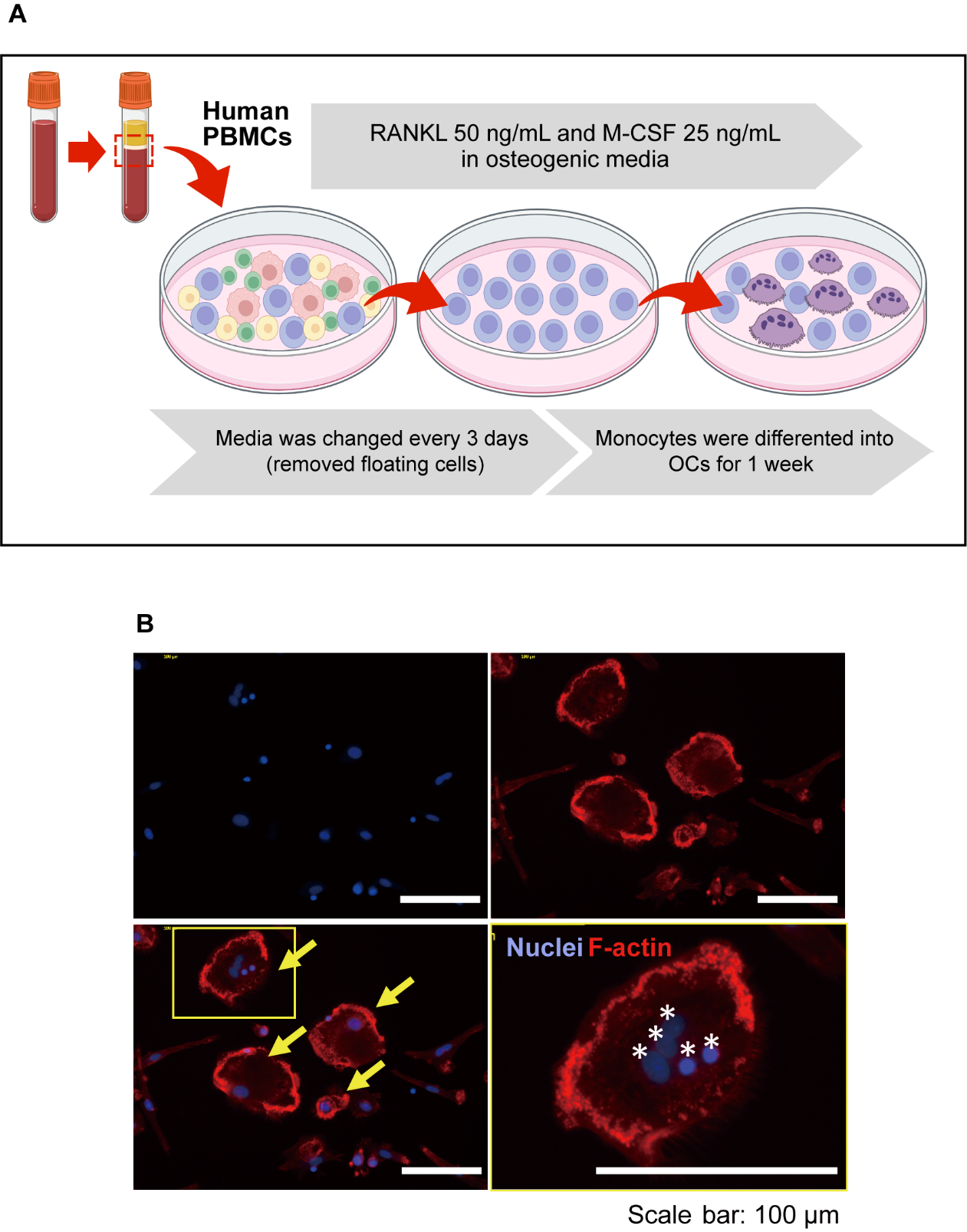


Fig. S6. Optimization of OC differentiation from human PBMCs. (A) Human PBMCs were cultured in osteogenic medium with RANKL (50 ng/mL) and M-CSF (25 ng/mL) to induce OC differentiation. The medium was refreshed every 3 days, with differentiation taking 1 week. (B) Representative images of OCs differentiated from PBMCs after 1 week, double-stained with Phalloidin 594 (F-actin, red) and Hoechst 33342 (nucleus, blue).

**References**

[1] Y.J. Yu, Y.H. Kim, K. Na, S.Y. Min, O.K. Hwang, D.K. Park, D.Y. Kim, S.H. Choi, R.D. Kamm, S. Chung, J.A. Kim, Hydrogel-incorporating unit in a well: 3D cell culture for high-throughput analysis, Lab Chip, 18 (17) (2018), pp. 2604-2613, 10.1039/c8lc00525g.
